# Expert recommendations on sharing medical information with patients: a qualitative study

**DOI:** 10.1186/s12913-025-13223-5

**Published:** 2025-08-28

**Authors:** Herman Egenberg, Hanne Cathrine Lie, Jennifer Gerwing, Julia Menichetti

**Affiliations:** 1 Department of Behavioural Medicine, Institute of Basic Medical Sciences, Faculty of Medicine, University of Oslo, Blindern, P.O. Box 1111, 0317 Oslo, Norway; 2 Health Services Research Unit (HØKH), Akershus University Hospital, Lørenskog, Norway

**Keywords:** Clinical communication, Health literacy, Medical information, Expert interviews

## Abstract

**Background:**

Sharing medical information with patients is essential for patient-centered care, yet empirical research to guide information sharing in clinical practice is inconsistent and scattered across disciplines. Clinicians rarely use established models for sharing information, and patients inconsistently understand and remember the information shared.

**Objective:**

Explore experts’ views on the task of sharing information.

**Design:**

Qualitative study using semi-structured interviews and reflexive thematic analysis.

**Participants:**

We recruited fifteen expert clinical communication teachers from six countries, using the snowball method.

**Approach:**

Interviews were recorded, transcribed, and analysed by authors with both clinical, teaching and research experience, using reflexive thematic analysis.

**Results:**

We conceived four themes addressing the task of sharing information. The overarching theme was: (1) Sharing information with patients should be a dialogue, not a lecture. Further, to improve how they share information with patients, clinicians might want to: (2) Help the patient process emotions; (3) Explore the patient’s knowledge and perspective; (4) Tailor and structure the information. Each theme included common challenges and solutions for clinicians.

**Conclusions:**

The findings align with and expand current models for communicating with patients. The themes integrate knowledge from different disciplines, such as psychology, medicine and communication science. The findings provide support for the role of information sharing in patient-centered care and shared decision making. The findings can guide clinicians in the task of sharing information with patients and shape curriculum and training development.

**Supplementary Information:**

The online version contains supplementary material available at 10.1186/s12913-025-13223-5.

## Introduction

Clinicians share medical knowledge in almost every consultation. Patients need to be informed about their condition, test results, treatment options, risks, benefits and follow up [1]. They require explanations of terms, tailored health advice, and help in navigating increasingly complex health care needs and systems [2].

At a policy level, governments around the world recognize the crucial role clinicians play in communicating medical information to patients and strengthening their health literacy [3]. Moreover, access to information is considered an important patient right in many countries [4, 5]. Therefore, part of providing good health care is being able to communicate accurate, relevant information and empower patients to make decisions aligned with their values and circumstances [6].

Sharing medical information with patients is a crucial, but complex and challenging task for clinicians. Several studies have shown that patients tend to forget or not understand 40–80% of the information provided in clinical consultations [7–9]. Patients’ poor recall and understanding of medical information can have an impact on health literacy, thereby reducing their ability to engage with medical advice and treatment decisions [10]. Low health literacy and low engagement with medical advice and treatment are currently a major healthcare concern [1, 11, 12].

While there is a need to improve information sharing practices [13], in general, translating research into practice is challenging [14]. In particular, the effort to improve information sharing practices by focusing on the communication skills is in its infancy [15]. One of the reasons is the lack or accessibility of research evidence. Research on how to provide medical information to patients is of interest to several disciplines, such as health literacy research, medical education, clinical communication, general communication science and pedagogy, each of which has developed in its own silo, with little or no integration [16]. A large scoping review of more than 9000 articles identified at least 19 different types of communication strategies for sharing information with patients, usually not informed by theory [17]. This fragmentation of the field might hamper implementing effective strategies in clinical practice. Indeed, even if some information sharing strategies such as structuring the information and asking patients to “teach-back” the information have shown promising results [18–21], clinicians rarely adopt these strategies in a meaningful way [22, 23].

To close the gap between knowledge and practice, there is a need for studies condensing and integrating theory-based and practice-relevant knowledge on how clinicians can effectively share information with patients during clinical interactions. Interviewing experts in a field is recognized as a fruitful way of providing deeper understanding of complex issues [24]. Experts can also work as knowledge hubs that can help connect overlapping fields and ideas [24]. Qualitative research can improve understanding of phenomena and is useful for providing depth and integration to fragmented fields of research [25]. As such, this study aimed to explore the views of expert teachers of clinical communication on the complex task of sharing information with patients. In particular, we aimed to:

(i) Synthesize in-depth understandings of information sharing;
(ii) Identify possible barriers and challenges to information sharing;
(iii) Provide actionable recommendations for clinicians.

## Methods

### Study design

This is a qualitative study using individual interviews with expert teachers of clinical communication. We approached our research from the ontological stance of constructivism, recognizing that the reality of clinical communication is shaped by the diverse experiences and perspectives of individuals. Epistemologically, our study is grounded in an interpretivist approach [26].

### Participants

We recruited fifteen “experts”, which we defined as clinical communication skills teachers with more than ten years of experience in teaching, and whom peers recognized as expert researchers in the field. The participants were approached by email and, if interested, signed a consent form with information about the study. We recruited the first three experts from the national collaboration network of the authors. Using a snowball method, we then asked for suggestions for further participants to interview (“Who do you think we should interview next?”), that would fit the criterion of being an experienced teacher of communication skills and a prominent researcher. In total, we approached 22 experts. Two had health or practical reasons for not participating and five did not respond to the invitation. The final group of experts had diversity in gender, professional background, and country in which they were teaching. They all had experience doing research in the field of clinical communication, and at least ten years of teaching experience. They taught medical students at different levels, as well as nurses, postgraduate doctors and specialists. See Table 1 for the participants’ characteristics.

**Table 1:** Participant characteristics

Number of participants	15 (contacted 22)
Nationalities	6 (Norway, Denmark, United Kingdom, Netherlands, USA and Australia)
Gender	9 males, 6 females
Average age (range)	59 (40–74)
Average years of clinical communication teaching experience (range)	23 (13–42)
Average years of clinical experience of experts who also worked clinically (5 of the experts had no clinical experience) (range)	25 (14–39)
Average number of citations (sources: Google Scholar, Researchgate, Scopus) (range)	11,159 (100–75 000 citations)
Professional background	medicine (7), psychology (2), nursing (2), anthropology (1), communication science (1), behavioral science (1), literature /philosophy (1)

### Reflexivity

The lead author HE is a PhD-candidate, medical doctor, and clinical communication teacher with 5 years of experience in teaching communication skills to medical students. JM is a psychologist with 11 years of experience in research on clinical communication. HCL is a psychologist and the head of clinical communication teaching at a Medical University Faculty. JG is a psychologist with 17 years of experience in research and training in clinical interactions and 25 years on basic research on face-to-face dialogue. HE conducted the interviews in parallel with teaching medical students how to share information with patients, carrying with him a strong interest in bridging theory and practice in clinical communication, shared by the research group. Rather than supressing this perspective, we regarded it as an analytic resource that shaped the questions we asked during the interviews and the direction of the analysis towards actionable suggestions. We engaged in regular team discussions to challenge our assumptions. For example, the first author’s experience may have initially emphasized educator-focused themes—but iterative reflection and co-coding with JM and HCL helped ensure that themes authentically reflected participants’ perspectives. HE corresponded with several external researchers experienced in qualitative research, presenting preliminary findings and discussing insights with the aim of improving validity. JG emphasized connections with contemporary communication theory and provided a bird’s eye perspective throughout the writing process. HE presented preliminary versions of the study in three different PhD-courses as well as at one national and one international conference for communication in healthcare and received wide-ranging feedback from peers. HE kept a reflexivity journal throughout the study.

### Procedures

When preparing for the interviews and creating the interview guide, we drew on the methodology “interviewing experts” [27] and Döringer’s work on implicit expert knowledge [24]. We chose to use a semi-structured interview guide (see Online Appendix 1). The main questions in the interview were “How should clinicians share medical information with patients?”, “What do clinicians and students usually struggle with?” and “How should information sharing be taught?”. While the scope of the study included both how to “do” the task of sharing information (best clinical practice) and how to “teach” information sharing (educational strategies), we found that the material on each topic was sufficiently rich to warrant separate attention. For this article, we focus solely on best practices for clinicians. The interview guide was revised several times in response to insights gained. For example, after one of the experts described how they used metaphors when teaching information sharing, the question “Do you use any metaphors when teaching information sharing?” was added to the interview guide for the subsequent interviews. We assessed the richness and diversity of the data in relation to the study’s aims. While the interviews were conducted, HE, JM and HCL reflected on whether the dataset was sufficiently information-rich to support meaningful theme development by asking reflective questions such as “Are we still learning something new?” and “Are we engaging with the data, or simply confirming what we already know?”. After 15 interviews, we decided that we had a sufficiently rich dataset. All the interviews were conducted and recorded using the digital platform Teams. Each expert was interviewed once, and the interviews lasted from 34 to 78 min.

### Analysis

We used Braun and Clarke’s protocol for reflexive thematic analysis [28]. HE conducted all the interviews and transcribed them manually using the f4Transcript software. HE then generated codes based on the first three interviews using the software NVivo. The coding style was then discussed with HCL and JM, refined and applied to the rest of the interviews by HE, with an iterative process of discussion and refinement. Initial themes and subthemes were generated by HE, shaped in the tension between the words of the experts and the purpose of the study. Initial themes and subthemes were discussed and revised with HCL and JM in several meetings. Through an iterative process, going back and forth between the transcripts, the codes, and the themes, HE and JM further developed the themes. After a discussion with all the co-authors and two external clinical communication scientists, the themes were refined. Labels for themes and subthemes were reviewed and revised to represent the gist of meaning. If possible, the experts’ words were used. At this point, we phrased the themes as “principles” and “key points”, while the subthemes were phrased as “challenges” or “solutions”. This framing of the themes and subthemes into a challenge/solution guide reflected the interview guide (e.g., *“*what are the challenges?*”*,* “* what do they struggle with?*”*, *“*what are the pitfalls?*”*). The themes and subthemes were then written out as a final report with supporting quotes and explanations.

## Results

“You must make your information giving much more frisbee than shot put… So you throw a little bit of information out… and then you get the patient to throw the frisbee back and then you know where to throw it next… it’s moving from this idea of “I’m only being helpful if I’m actually the transmitter.” (Expert 15)

We conceived four main themes regarding sharing information with patients: (1) “Sharing information with patients should be a dialogue, not a lecture” (2) “Help the patient process emotions” (3) “Explore the patient’s knowledge and perspective” (4) “Tailor and structure the information”. The first theme was a “higher order” theme, functioning as a guiding principle encompassing the others, while the others were considered key aspects of sharing information. Each theme included subthemes that related to specific challenges and solutions. Table 2 summarizes the four themes and related subthemes.

**Table 2:** Themes and subthemes

Themes	Subthemes
Sharing information with patients should be a dialogue, not a lecture *(guiding principle)*	Challenge: Clinicians are habitualized and expected to be a lecturing expert
	Solution: Strive for the goal of shared understanding
	Solution: Use established clinical communication models
Help the patient process emotions	Challenge: Emotions affect patients’ capacity to handle information
	Solution: Pay attention to the emotion
	Solution: Respond with empathy
Explore the patient’s knowledge and perspective	Challenge: Clinicians wrongly estimate what patients understand, and why they do not engage
	Solution: Map patients’ preferences and knowledge
	Solution: Use “teach-back” to ensure shared understanding
Tailor and structure the information	Challenge: Clinicians tend to overwhelm patients with too much, too detailed and too complicated information
	Solution: Tailor
	Solution: Provide structure

### The guiding principle: sharing information with patients should be a dialogue, not a lecture

As all experts pointed out: *“information giving is an interactional process” (expert 10).* The experts reflected on this point as being an underlying, guiding principle:

“One of the biggest principles of information sharing is that it is a dialogue, not a monologue.” (expert 05)

The experts contrasted sharing information in a dialogue with outdated “sender-receiver” models of communication, where information sharing is seen as a linear process where one “downloads information” from the other (expert 02). A striking analogy for these contrasting models is that bad information sharing practice looks like throwing “shot put” at the patient while good information sharing practice looks like throwing a *“frisbee”* back and forth *(expert 15).*

#### Challenge: clinicians are habitualized and expected to be a lecturing expert

Some of the experts explained why it may be hard for clinicians to share information with patients in a dialogic way, and why they can often end up speaking to the patient as if they were delivering a lecture. They discussed that professional identity built during clinical training, and maintained during clinical practice, could hinder clinicians in adopting a dialogic approach with patients. One expert mentioned that clinicians are trained to be knowledgeable and to help by providing answers and solutions to patients.

“It is moving from this idea of I’m only being helpful if I’m actually the [information] transmitter.” (expert 15)

One expert reflected further on how students are taught in a lecturing way during medical school, implying that clinicians may adopt this approach when sharing information with patients later.

“In the first couple of years of medical school, the only way they learn is by getting information.” (expert 02)

One expert mentioned that patients also reinforce this position, as they expect the clinicians to be knowledgeable and trustworthy experts. Breaking these expectations, by for example opening up about uncertainty, can be challenging.

“The medical practitioner represents a stereotype, a cultural, archetypical persona. So there is a large degree of trust in that archetype… So we play on that, so trust can be established very swiftly. Because you are THE doctor. And many times, what is most important is that you behave in accordance with people’s expectations, enough for them to maintain this trust.” (expert 06)

One of the consequences of this “expert identity” is that clinicians may see uncertainty and lack of knowledge as a threat, as something to avoid. Uncertainty and fear may lead to lecturing about the information, without giving space for questions or other input from the patient.

“If the doctor is very uncertain about the information he or she should provide, they tend to talk more.” (expert 12)

“They are scared of getting questions they don’t know the answers to. So they don’t leave gaps for the patient to ask.” (expert 09)

#### Solution: strive for the goal of shared understanding

According to the experts, clinicians should move from the old-fashioned goal of *transmitting* information from one person to the other to deliberately set a goal that orients to the other person. Instead of “have I made myself clear” the goal should be “do we understand each other”.

“The information giving is not so interesting. It’s the recipient’s ability to make sense of this information and turn it into something of value that is interesting.” (expert 06)

In this regard, many of the experts used the term “shared understanding”. Shared understanding means that both parties in the conversation co-create meaning in the dialogue. That is, they learn from each other, step by step, and adjust their words and actions incrementally, until they are satisfied that “I know what you mean, and you know what I mean”. By viewing shared understanding as something that is co-constructed, it is easier for the clinician to move from being a lecturer to being in a dialogue with the other person.

“I have absolutely switched (from using the term giving information) to sharing.” (expert 05)

“You work together to find that common ground, that kind of shared understanding.” (expert 07)

By aiming for shared understanding, experts suggested that clinicians can bypass many of the challenges of “giving” information. Having this goal consciously in mind helps the clinician communicate in a way that serves the patient.

“It (sharing information) is not about you (the clinician).” (expert 15)

#### Solution: use established clinical communication models

All the experts indicated that using established clinical communication models, guides or techniques can help when sharing information as part of a dialogue.

“For you to be a good communicator as a doctor, you have to have a toolbox of communication resources that you can draw on.” (expert 07)

One expert pointed out how too many clinicians were *“totally winging it” (expert 04)*, meaning that clinicians tend to rely on their natural communication skills developed through everyday life, rather than considering clinical communication as a professional skill that ought to be learned.

The experts referred to several useful models and methods. Several experts mentioned that they use generic clinical communication guidebooks such as the Calgary Cambridge Guide and the Four Habits Model that include strategies for information sharing.

“We tend to stick to that model [Calgary Cambridge Guide], but in the postgraduate teaching we’ll talk about other models, for example the Four Habits.” (expert 15)

Other experts mentioned other more specific resources for focused information sharing tasks. One expert, for example, said: *“in the context of breaking bad news*, *I would use the SPIKES model” (expert 11)*. Another expert endorsed this model for its ability to guide clinicians to be mindful of the patients’ emotions and perspective.

“And what I liked early on about SPIKES is that it includes empathy, the E stands for empathy.” (expert 08)

Many experts referred to Ask-Tell-Ask and Chunk-And-Check, as communication formulas that are easy to remember and apply. With Ask-Tell-Ask, clinicians are guided to involve the patient by “asking” the patient for their pre-existing knowledge, preferences, and understanding as a part of the “telling” process. Such involvement would create a more dialogic, back-and-forth quality of the interaction.

“I very much like this slogan so to speak from American oncology, ask tell ask, indicating that to TELL, giving information, is given in a context of asking, listening. So I think one of the most important principles is that information giving is part of an interactional process that includes, in a way, always asking the patient, or listening to where the patient is, before you give information. And when you give information, kind of test that out while you’re listening to the patient’s response.” (expert 10)

With Chunk-And-Check, clinicians are encouraged to share information in appropriately sized “portions” to avoid overwhelming the patients, and regularly check if the patients have understood.

“Give small blocks of information and then check the understanding along as you give it.” (expert 13)

Finally, two specific techniques were mentioned as useful tools for sharing information. One was the newspaper or book metaphor for structuring information and the other was the teach-back method for checking understanding. These will be further explored in theme 4.

### Help the patient process emotions

Many experts emphasized the importance of helping patients work through the emotions that might arise when sharing information. It involves anticipating, recognizing, and making space for emotions in the consultation.

#### Challenge: emotions affect patients’ capacity to handle information

As an expert emphasized: *“it’s always that way: Emotions trump facts” (expert 06)*, meaning that the patient’s emotional state and/or reaction has a powerful influence on the information sharing process. This mechanism was mentioned by many of the experts, with one using the term “arousal”.

“Arousal stands in the way of cognitive processing.” (expert 03)

Some experts pointed out that medical information is intrinsically charged with emotions, with different degrees of intensity. Furthermore, medical information might come in conflict with patients’ previous expectations, understanding or needs. Therefore, even information that might seem neutral or innocuous to the clinician might cause emotional responses such as fear, anger or confusion for the patient.

“Do not think information is free from affect. When you go over your test results with patients, they’re going to have an affective reaction to what you tell them.” (expert 07)

As a consequence, the emotional response may vary greatly and “*the information can be received completely differently by patients depending on the level of emotional charge that goes with it*” *(expert 04)*.

Anticipating and making space for emotional responses are especially important when breaking bad news. Sometimes information can be a matter of life and death, for example, when breaking the news that the patient has terminal cancer.

“Everything you say after you have given the serious news, the patient will tend to not remember.” (expert 10)

In such cases, the emotional responses the patients have may greatly hinder the process of sharing information and the patients’ understanding, especially when the clinician does not manage to address the emotions appropriately.

“They didn’t address the emotion in the room, and because they didn’t address the emotion, the information went right past them.” (expert 04)

One expert mentioned that “feeling unsafe” can become an emotional barrier to processing the information.

“If the patient doesn’t feel safe with you, it will be hard for the patient to follow information that is cognitively loaded.” (expert 12)

#### Solution: pay attention to the emotion

One expert specified that in order to help the patients process their emotions, the clinician must first become aware of them. This includes paying attention to, and actively searching for emotional responses.

“You have to pay attention to the emotion.” (expert 04)

Reflective questions can help remind the clinician to consider the inner emotional world of the patient.

“What does this person feel like at the moment? Does he feel threatened by some ominous disease or looming death? Or does he feel bewildered?” (expert 06)

Several experts pointed out that clinicians might need to adjust their view of emotions and of emotional responses. Emotions are not something to oppose or to battle, not something dangerous, but rather something natural to accept and make space for.

“I try to tell my students that crying is not dangerous.” (expert 08)

“If the patient is upset, that actually means they understood the information.” (expert 04)

Thus, having an accepting and curious attitude towards emotional responses might be a first step in helping the patients process their emotions.

#### Solution: respond with empathy

Several experts noted that addressing patients’ emotional responses is an important part of sharing information.

“If the emotion is significant, we need to address it.” (expert 04)

Several experts emphasized the importance of empathetic responses. As one expert noted: *“Empathy is about stating your witnessed emotions. You have to say something” (expert 05)*.

Addressing emotions can be done in several ways and benefit from different strategies.

“We experiment with several techniques, nonverbally, putting a hand on the shoulder, pushing a Kleenex box in the direction of the patient, to more verbal responses like: I know this is not the news that you wanted.” (expert 08)

One expert suggested to *“respond in a way that acknowledges the patient’s emotions” (expert 10)* pointing to validating emotions as an important element in information sharing.

When clinicians not only accept emotions but also validate them, the patient might have the opportunity to process the emotions, thereby being more able to process the information that is being shared.

### Explore the patient’s knowledge and perspective

With this theme, the experts stressed how checking and exploring the patient’s perspective throughout the consultation can help the clinician make sure they are sharing the right information at the right time and at the right level, in accordance with the patient’s preference and values. Patients’ perspectives can include their previous knowledge about the topic, information needs, expectations and emotions. Exploring each patient’s individual perspective is a natural step towards sharing information in a dialogic fashion.

“One of the key skills is starting by saying: We think you have diabetes; what do you know or what do you think about it?” (expert 05)

#### Challenge: clinicians wrongly estimate what patients understand and why they do not engage

According to the experts, a common mistake of clinicians is failing to grasp how much the patient understands about their health, their condition and their treatment. As a result, it is difficult to find common ground for building incremental knowledge during the information sharing process.

“I did some work with a sociolinguist a few years ago where we took little pieces of the encounter and noticed how the clinician and the patients were like ships passing in the night; they just didn’t really understand each other.” (expert 01)

“It’s so incredibly important to match the patient in terms of their health literacy.” (expert 15)

Wrongly estimating the patients’ level of understanding may be due to clinicians being unaware that they are more experienced and knowledgeable about medical information than their patients are. One expert pointed out how clinicians might be interacting predominantly with other medical professionals and highly educated people in their daily lives, thus skewing their expectations of their patients’ health literacy.

“Even in the first year, they (medical students) are in the top 10% of the country in terms of knowledge. And I think getting them to understand that the rest of the country are not like that, you know, people don’t understand medical terms.” (expert 09)

Furthermore, some patients tend to be reserved and quiet during consultations, which can be indicated by, for example, not interrupting or speaking up when they do not understand something. One expert argued that this apparent reticence is partially because of the power differential, of which clinicians might be less aware.

“Keep in mind that often patients will say yep, I understand, but there are lots of barriers to them actually admitting that they don’t.” (expert 11)

“They (doctors) don’t understand how difficult it is to interrupt a doctor because of the power differential.” (expert 12)

#### Solution: map patients’ preferences and knowledge

The experts claimed that in order for clinicians to share information effectively, they need to “*assess the starting point*” *(expert 05)*, which includes to *“check what the patient knows from before” (expert 10)* and ask *“what have other physicians said?” (expert 10)*. Mapping the patient’s preferences and knowledge can save time and effort and help the clinician tailor the information to that particular patient (see theme 4 for more details about tailoring).

“Because so much of effective information sharing is finding out where the patient is at, what they know, and what they’re worried about.” (expert 05)

“Your choice of the way you inform will depend on the level of understanding and information the patient has beforehand.” (expert 10)

Several experts emphasized the importance of finding out what and how much the patient actually wants to know. Regularly assessing the patient’s preferences and previous knowledge requires, according to the experts, that clinicians ask questions throughout the consultation, also as a part of the process of sharing information.

“You should be asking questions as part of the giving information process. So you’re making sure: what does the patient know already? What does the patient want to know? Is the patient following what you’re saying?” (expert 08)

Patients’ preferences for information vary. As one expert put it:

“Some patients don’t want to know at all… and unless you find that out, your explanation is going to fail.” (expert 09)

The only way to find out for sure is to engage the patient. The experts proposed several ways of encouraging engagement while sharing information: using pauses and silence, providing a question prompt list, and using meta-communication.

“Without saying it, I’m using a pause to give the patients the possibility of asking.” (expert 12)

“One of the easy but often researched and proven effective interventions on the part of patients is the use of question prompt lists. Basically it’s a list of questions patients can look at in preparation of a consultation.” (expert 03)

“I’ll explain a little bit, but you know, stop me if I’m telling you something that you know, feel free stopping me with questions.” (expert 11)

Paradoxically, asking the patient “Do you have any questions?” could be a suboptimal way to encourage questions, because the patients will often reflexively answer “no”. As one expert put it:

“Asking “any questions?” with an upward tone on your voice means I’m done, and I hope you don’t have any questions.” (expert 01)

#### Solution: use “teach-back” to ensure shared understanding

Several of the experts recommended applying the principles of teach-back to ensure shared understanding. Teach-back is a method where the clinicians would ask the patient to say back the information they have talked about in the consultation. If a patient understands, they are able to “teach-back” the information accurately.

“Several years ago, I had a patient with diabetes, his HbA1C was high. He was on Glipizide at the time, so I said, “I’d like you to add Metformin twice a day.” And then three months later he comes back and his HbA1C is still 8,5 and I say “Ok, any problems taking the Metformin?” And he goes “No, I’ve been taking that new medicine, it’s not bad.” “Ok so you’re taking Glipizide and Metformin?” And he goes “Oh, I thought you wanted me to stop the other medicine.” So, all of a sudden, we have lost three months of potential diabetes control… and I think that’s where teach-back could have really been helpful.” (expert 02)

However, the experts also mentioned that teach-back is not commonly used.

“Even though it (teach-back) is a good idea, I’d say maybe 20% of all the doctors in the world use it.” (expert 05)

Indeed, as many experts said, teach-back might feel awkward if not presented to the patient in a thoughtful way.

“I think there’s another barrier to teach-back, and that is we sound condescending. Tell me what I just told you.” (expert 02)

The experts suggest phrasing the request for teach-back in different ways to avoid feeling awkward.

“In the end, you must have sufficient time to ask… without giving the patient a feeling of being examined: I know I’ve given you a lot of information now, and it’s important to me to know what you take back from this.” (expert 12)

“The other approach to this is: I wonder what you will tell your loved one when you go home.” (expert 02)

One expert pointed out that by experimenting with teach-back, clinicians might experience its importance, thus making the practice of using teach-back self-reinforcing.

“Actually, the doctors I’ve seen who use it the most are the ones who try using it… they’ll say: So, what’s your main understanding of what you’re going to do when you go home, and the patient is like: Wrong. And they realize they’ve just spent 10 minutes explaining something, and the patient didn’t follow it at all. And that’s when they start using it regularly.” (expert 05)

### Tailor and structure the information

Many of the experts alluded to a style of information sharing where the clinician takes the role of a teacher or a facilitator, rather than just an expert who is sharing their knowledge with the patient. They advocated an approach to sharing information that requires structure and tailoring.

“The understanding is through the interaction, not just through the telling.” (expert 07)

#### Challenge: clinicians tend to overwhelm patients with too much, too detailed, and too complicated information

Several experts highlighted that clinicians tend to overwhelm patients with information.

“So, what I find is that when I work with doctors there seems to be something about informing too much, giving too much information still. And repeating their information too many times instead of checking in with the patient what their perspective on what they have just heard is.” (expert 14)

“Clinicians often speak for a very long time with no interruptions, and they become very unaware that they are talking “medicalese”, using medical jargon terms.” (expert 01)

One expert remembered observing a medical student who *“in one sentence without pausing for breath*,* she gave 9 pieces of information to the patient” (expert 09).* Another expert provided a concrete indication of what may be “too much” information.

“If you’re speaking for more than three sentences at a time, it’s probably too much information.” (expert 02)

One expert noted that this may be due to not being able to see things from the perspective of the patient.

“One of the reasons that they overload is that they still find it hard to put themselves in the position of the patient." (expert 03)

#### Solution: provide structure

For several experts, one key solution for avoiding overwhelming the patient with too much information was working on the structure of the information sharing, in order to make it easier for the patient to follow and remember. They gave several tips for how to structure information. For example, some suggested signposting, meaning to signal to the patient what kind of information is coming up.

“There’s a small one which sometimes is very helpful: signposting or saying. “Well, I’m going to talk to you about this, this and this.” So, they can follow what you’re saying… You can even use your fingers.” (expert 05)

One expert suggested using a headline, like in books or newspapers, to set the stage for the information sharing session.

“You can tell the rest of the story, but you need to start with a headline, so the patient knows what you’re talking about instead of telling them the story of how you got to the diagnosis. And that’s the big error that I see untrained physicians make over and over again… You can go into the other stuff, but the first part is: Your culture showed you have strep throat, period. One sentence, no clauses, stopped at the end.” (expert 04)

This kind of structuring can be challenging, especially if the clinician is unprepared for the conversation. A good start might be to ask oneself reflective questions that orient to what the patient really needs to know and what the clinician is trying to achieve.

“You should ask yourself the question: what exactly does this patient need to know NOW. That should guide you in prioritizing which information to bring forward.” (expert 12)

“Most of the time, I think students need to think: What am I doing here? Am I instructing, explaining or comparing? And how much depth do I need to go into?” (expert 01)

A final point on structure was managing the tempo and using *“the fantastic efficiency of small pauses.” (expert 12).*

“The other thing that’s incredibly important is to pause, because I think pauses are actually an opportunity for clarification.” (expert 01)

#### Solution: tailor

Many of the experts noted the importance of tailoring the information to each individual patient.

“It’s no point having a script: Like, this is the information I want to give everyone coming for bowel screening.” (expert 15)

One expert used the term “bespoke”, meaning *made for a particular person.*

“I think it’s really important to start with their [the patient’s] agenda rather than say the same things to everybody. It makes it [the medical information] bespoke. And so, I say to the students: you need to make your information giving bespoke rather than standard. What is it that this individual needs?” (expert 15)

Letting the patient drive the information flow can require some patience and self-discipline, as it can be tempting for clinicians to just explain everything they know.

“I encourage the students to be patient. The patient will get there. Answer the questions you’re asked. Keep it short and simple, and let the patient control the amount and the pace of the information giving.” (expert 08)

Tailoring requires engagement, which ties back to the previous theme of exploring the patient’s perspective and knowledge. One expert noted how the level of shared understanding is a product of how much the patient talks during the consultation. With a better understanding of the patient’s perspective and knowledge, tailoring follows naturally.

“[Shared understanding] is greater when you get the patient to talk more about what they think, what they want, what they believe.” (expert 07)

## Discussion

This study aimed to explore expert views on the task of sharing information with patients. We conceived one general principle: (1) “Sharing information with patients should be a dialogue, not a lecture,” and three supporting key points (2) “Help the patient process emotions,” (3) “Explore the patient’s knowledge and perspective,” and (4) “Tailor and structure the information.” Each theme included common challenges and possible solutions.

### A conceptual model for the task of sharing information with patients

**Fig. 1:**
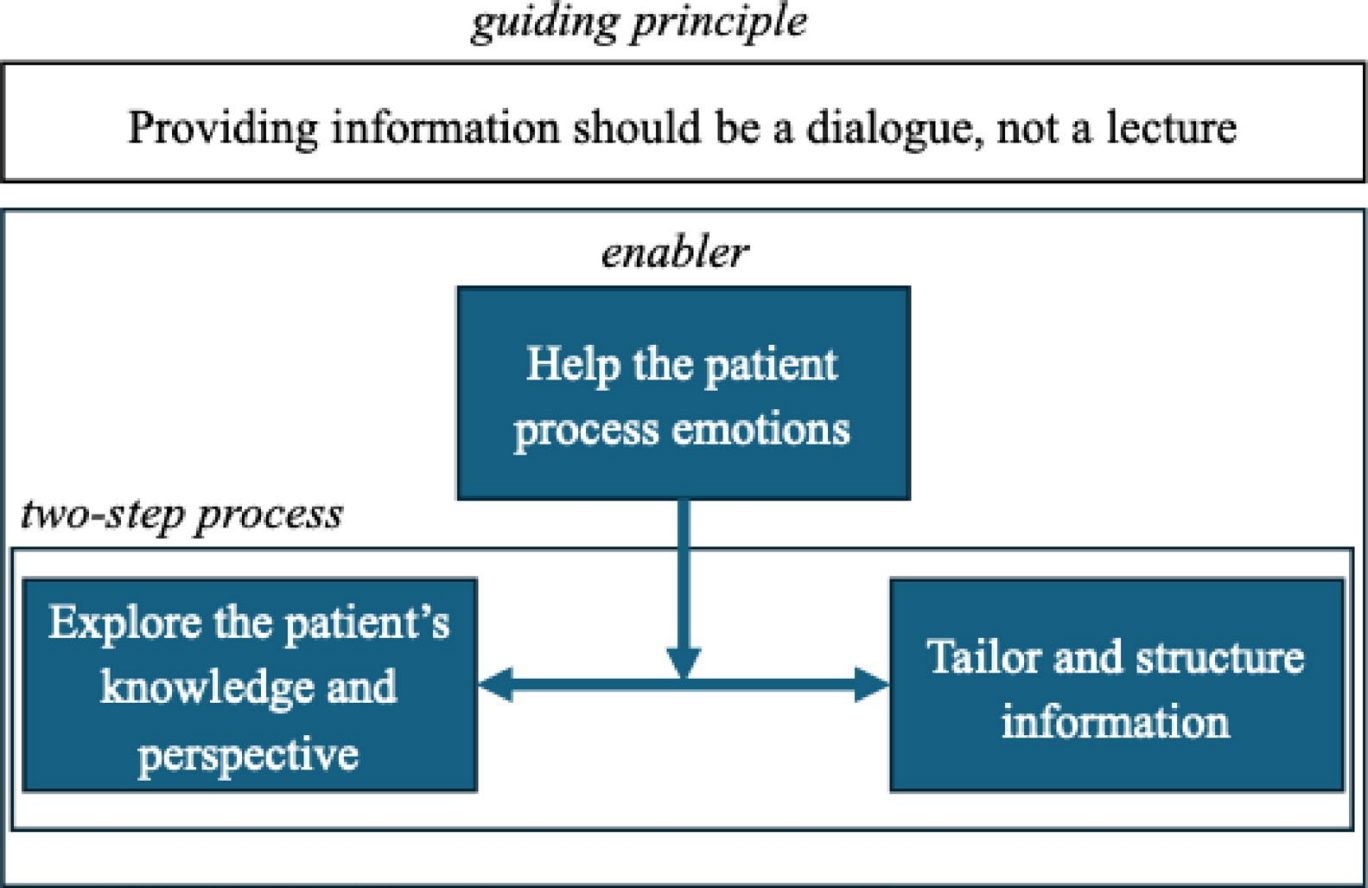
A conceptual model for the task of sharing information with patients

We presented the first theme as an overarching guiding principle, while the other three themes represented more concrete, interconnected actions for realizing the guiding principle. “Help the patient process emotions” functions as a premise for enabling dialogic information sharing, while “explore the patient’s knowledge and perspective” and “tailor and structure the information” are interdependent parts of a process: one requires the other to be successfully realized. Figure 1 offers a conceptual map of the four themes.

For a patient to understand and retain information, they may need the clinician to provide an opportunity to process emotions that come up. Indeed, several studies from different fields underscore the effect emotions have on learning, cognitive processing and memory [29, 30]. While the role of emotions has been recognized in the specific task of breaking bad news to patients [31], the experts emphasized the importance of emotions when sharing all sorts of medical information. This distinction between “bad” news and other information might be helpful in some clinical situations. However, it might be difficult for a clinician to predict which news is “bad”. For example, a clinician might consider a blood test showing normal blood results as good news, but the patient might be upset, because they had hoped the blood results would explain their symptoms. Therefore, clinicians should be prepared to pay attention to the patient for signs of an emotional response every time they share medical information. In this regard, empathic skills have been emphasized by experts as crucial for good information sharing. Indeed, previous studies have shown how empathic information sharing can increase patient understanding of the information [32, 33]. Since emotional and information sharing processes in clinical consultations have tended to be explored in isolation from each other, further studies may be needed to understand how communication behaviors, emotions, and cognitive processing are linked.

Similarly, the interaction between theme 3 (“Explore the patient knowledge and perspective”) and 4 (“tailor and structure the information”) is also recognized in recent understanding of information tailoring [34] and modern healthcare models e.g. evidence-based medicine, patient-centred care, and shared decision-making [35]. Indeed, in shared decision-making, patient values and preferences are explored after explaining treatment options (with information tailoring often mentioned), often based on patient knowledge [36]. A systematic review from 2022 [37] showed that a congruent approach to caring for patients requires a connection between the patient’s values and preferences and what the clinician can share from their knowledge and experience, something that inherently involves information sharing. However, to the best of our knowledge, ours is the first study highlighting how this two-step, interconnected process of (1) exploring and (2) tailoring with structure in the information sharing, enabled by emotion management, can contribute to a dialogue-oriented information sharing. Albeit dialogue is a core concept in the model of patient-centered care [38] and in the concept of shared decision- making [36], previous studies in the clinical communication field have not detailed what exactly “dialogue” entails. If we move to basic research that has tested models of how face-to-face interaction works, research has moved from the obsolete *message-receiver model* to a *collaborative grounding model*, where people build shared understanding through ongoing interaction and mutual adjustments during conversations [39]. By doing so, the clinician understands the patient’s perspectives better, while simultaneously, the patient understands what the clinician is talking about better. Thus, the experts’ views align well with contemporary communication models that attempt to describe how dialogue functions.

### How the model connects to other clinical communication models

In general, the four themes align with already established patient-centered communication skills training models such as the Calgary Cambridge Guide [40] and The Four Habits Model [41]. This result is not surprising, since many of the experts actively used these models in their training.

The Calgary Cambridge Guide is an in-depth guide to the medical consultation from start to end, offering a clear structure to the consultation. The Four Habits Model is a simpler model for patient-centered communication in medical encounters. It encourages four independent communication “habits” with their family of skills: invest in the beginning, elicit the patient’s perspective, demonstrate empathy and invest in the end.

A key difference between these models and our findings is that existing models are much broader in scope, encompassing all aspects of clinical communication and often include a standardization of the consultation process, while this study reveals the deep mechanisms and intricacies of the specific clinical task of sharing information with patients. Specific techniques for sharing medical information with patients, such as Ask-Tell-Ask [42], Chunk And Check [43], the SPIKES model [31], and the teach-back method [44], reflect an unmet need of specifically addressing information sharing and have indeed been highlighted by the experts. However, our findings reflect a wider view of the task of information sharing, where these formulas represent possible pieces but not the whole picture. Indeed, the goal highlighted by the experts in our study (strive for shared understanding) extends some of the goals of these formulas. Accomplishing shared understanding indeed represents a bigger and more dynamic endeavor than seeking confirmation for patient understanding with e.g. teach-back. Such a difference is clearly reflected in the first theme of our findings (“Sharing information with patients should be a dialogue, not a lecture”).

In a way, our findings more closely align with empirically-derived models of communication, which focus more on the process of the clinical interactions rather than on the structure [45]. According to such models, a patient’s emotional expression triggers an opportunity for the clinician to open up a dialogue and meet the patient. While these models mainly focus on the emotional and empathetic parts of a conversation, our findings align with their overall pattern of cycles of attention to the patient, followed by an adjusted response. Our findings also highlight how emotions and information-sharing interact, and shift the goal from simply making the patient feel understood to creating mutual understanding, which is similar to models of empathetic communication [45].

Finally, our study provided in-depth experts´ views of the task of sharing information with patients and actionable recommendations for clinicians. Our study included insights about possible barriers and challenges to why achieving dialogue with patients may be difficult, and why clinicians might resist dialogue, which is something that the traditional models does not solve. In the next section, we will discuss possible, specific directions for how to address such challenges based on our findings and previous literature.

### Implementation paths

The experts proposed that one challenge of adopting a more dialogic style of communication is that clinicians are used to conventional lecture-based learning from their training, and therefore become naturally inclined to lecture their patients in a monologic fashion. Indeed, lecture-based learning has been and is still the dominant way of teaching medical students [46]. In education, students have a tendency to mimic what their teachers and role-models do, over what they say [47]. This tendency could suggest the need for more interactive, dialogue-oriented training, coherent with how clinicians are expected to interact with patients.

The experts also pointed to identity-related, psychological barriers as a hinderance to being in dialogue with patients. That the clinician’s role or identity could steer them away from dialogue and shared understanding is aligned with previous studies [48]. Therefore, future communication training might generally build professionalism and a patient-centred professional identity, and could also improve dialogic information sharing [48]. Currently, professional identity formation practices in medical schools are scattered and lack consistency [49]. We suggest an increased attention to clinicians´ professional identity development and its conflicts and dilemmas during education (e.g., working on meta-skills and designing uncertainty tolerance training) as necessary for good information sharing [50, 51].

Then, according to the experts, clinicians commonly misestimate patients’ ability to understand, as well as their needs and preferences for information, and in general, tend to overwhelm patients with information. Misestimating the patients is possibly linked with cognitive biases such as the “curse of knowledge” which explains how it is difficult for humans to fathom that others might not know what they themselves know [52]. Such phenomena can substantially hinder the possibility for informed consent and patient involvement [53]. More customized information tools may prevent clinicians from relying on subjective speculations, for example, by using validated instruments to assess patients information needs or abilities or by training clinicians in recognizing how health literacy manifests communicatively [54]. Although teach-back was forecasted as a possible solution, the experts also noted its challenges, which are also reported in the literature [55]. According to our experts, rationale-based training may be preferred over performance-based, that is, clinicians (and patients) may be better served if the clinician understands the reasoning behind teach-back and is offered a variety of possible examples for how to achieve it [56]. Further studies may be needed to analyse the main causes underlying the low use rate of teach back and when (and how) clinicians and patients accomplish it successfully.

### Strengths and Limitations

This study contributes to a more integrated understanding of how clinicians might share information with patients by drawing on the combined expertise of experienced clinical communication educators. By involving experts who operate at the intersection of teaching, research, and clinical practice, the study bridges disciplinary silos and connects scattered knowledge across fields such as medicine, psychology, and communication science. Using a reflexive thematic analysis approach, we developed a nuanced account of the challenges and principles of information sharing, grounded in the lived insights of those who teach communication in diverse healthcare systems. The findings offer not only conceptual clarity, but also practical strategies that clinicians can apply in everyday practice.

One limitation of this study is the exclusive reliance on expert opinions, which do not represent the perspectives of patients or other healthcare professionals. Furthermore, the participants were from Western countries, and from the same scientific community, limiting the generalizability of the findings to other cultural contexts. Indeed, using a snowball method to recruit our participants risked gathering homogeneous views; however, clinical communication is a sufficiently broad topic that some diversity could be expected regarding their views of the specifics of information sharing. Furthermore, our definition of “expert” was somewhat time-based; thus, we were not able to single out clinical communication teachers whose views and methods had evolved. We mitigated these limitations by ensuring that our expert panel included individuals with diverse clinical and teaching experiences across multiple healthcare systems and who were recognized in the research milieu as well, which demands keeping up to date with contemporary models and state-of-the-art research.

Further research may complement this knowledge with clinicians’ and patients’ views, as well as views from experts from non-western countries. Future studies may test whether training based on these findings can be effective in increasing clinicians’ and patients’ shared understanding of information. Also, future studies could explore how these principles are applied in healthcare practice, including different cultural contexts. Further studies could finally address how the task of sharing information should be taught to students.

## Conclusion

When a clinician shares information in a lecturing style, the information is often generic and filled with too many details. The patient does not speak much and there is no way of knowing if the clinician and patient understand each other. On the other hand, when information is shared in a dialogic style, the clinician might pay more attention to the patient, ask questions, like “what do you know about this already?” and “what is important to you?” and tailor the information to what they learn about the patient’s needs. Whenever a new term is introduced, the clinician might ask “have you heard about that?”, and if the patient answers “yes”, they might ask how the patient understands the term. By listening, validating, and using what the patient says, the clinician encourages the patient to be active in the conversation. If emotions arise, the clinician will address them and hold space for them. Finally, the clinician will check that they have a shared understanding of the topic discussed.

The task of sharing information with patients is indeed complex, but it is of vital importance that it is done well. A deeper understanding of the intricacies of the task and clear recommendations for clinicians as provided here might have consequence for medical education, patient safety, and further research in doctor-patient communication.

## Supplementary Information

Supplementary Material 1.

## Data Availability

The anonymized interview transcripts generated and analyzed during the current study are available from the corresponding author on reasonable request.
