# Supplementary material for: Expert recommendations on sharing medical information with patients: a qualitative study
Source: BMC Health Serv Res. 2025 Aug 28;25:1137. doi: 10.1186/s12913-025-13223-5 (PMC12395779; doi:10.1186/s12913-025-13223-5)
Supplement: Supplementary file 1 — Supplementary Material 1. [file 12913_2025_13223_MOESM1_ESM.docx]

# Table of contents

- interview guide, final version
- interview guide, first version
- consent form
- qualitative research checklist

# (FINAL VERSION) Interview guide

The purpose of the expert interviews is to gather “know-how” from leading experts in teaching clinical communication, focusing on strategy grounded in theory, as well as strategies not yet described in theory. A synthesis of their wisdom, combined with empirical work, will be part of a scientific study, as well as inform the development of a new program for teaching information giving to medical students at the University of Oslo.

This semi-structured interview has a loose structure, putting a lot of responsibility on the interviewer to be able to fluidly follow up leads and direct the interview while interpreting answers on the spot. They should use active listening techniques, follow-up questions, reflections and silence as tools to enable the expert to talk freely and to get a deep understanding on their views on information giving. Following are the themes to be covered and example questions/prompts:

Before recording starts:

- What is your age
- nationality
- gender
- professional background
- years of experience as clinician, teacher and
- area of expertise
- other parallel activities such as teaching and research

Introduction: The interviewer should make the purpose of the interview clear; and introduce themselves as well as their knowledge and interests in the field.

Warm up questions. The purpose of this part of the interview is to establish trust and rapport between interviewer and expert, as well as to start breaching the subjects of information giving and teaching. The interviewer should add personal references and opinions where suitable.

- How did you end up working with clinical communication?
- Do you like teaching?

Personal opinion.

- Can you tell me where you teach information giving, and to whom?
- How do you like to define information giving?
- What is the most important things you want your students to learn about information giving?
- Which tips or tricks do you most frequently give to students?
- What do students usually struggle with when it comes to information giving?
- What do patients want from doctors when it comes to information giving?
- In your opinion, what are the most important skills of information giving?
- Do you use any metaphors, or games/exercises when teaching information giving?
- Do you use any clinical examples or stories in your teachings?

Theoretic framework.

- Is your teaching of “information giving” informed by any particular theoretic framework, and if so which one?
- Do you use framework or theory from pedagogics to inform the design of the curriculum and study program?
- Which books or articles do you want your students to read?
- What discoveries have shaped the way we think about information giving?
- In your opinion, who has been the most important expert to drive research and/or theory forward in this field?

Conclusion.

- What is your advice for us as we are about to create a new program for information giving for medical students in Oslo?
- What kind of set up is best suited for information giving? Lectures? Small groups? Roleplaying? Real patients?
- What are the pitfall of designing a course on information giving?
- Who do you recommend I interview next? Two or three names. Diversity.
- Is there anything else that I should have asked you about that you think is important?

## (FIRST VERSION) Interview guide for the study “Overview of current theory and teaching strategies on information giving. Qualitative study using in depth interviews of leading experts in Norway and internationally.”

The purpose of the expert interviews is to gather “know-how” from leading experts in clinical information giving, focusing on strategy grounded in theory, as well as strategies not yet described in theory. Finally, we will create a new program for teaching information giving to medical students at the University of Oslo using this study’s findings as guidance.

The semi-structured interview has a loose structure, putting a lot of responsibility on the interviewer to be able to fluidly follow up leads and direct the interview while interpreting answers on the spot. They should use active listening techniques, follow up questions, reflections and silence as tools to enable the expert to talk freely and to get a deep understanding on their views on information giving.

Introduction: The interviewer should make the purpose of the interview clear; and introduce themselves as well as their knowledge and interests in the field.

Warm up questions. The purpose of this part of the interview is to establish trust and rapport between interviewer and expert, as well as to start breaching the subjects of information giving and teaching. The interviewer should add personal references and opinions where suitable. Example questions:

- How did you end up working with clinical communication?
- What do you think about teaching?
- What is you your favorite part of your job?

Theoretic framework. Example questions:

- What is the theoretic framework you use for teaching information giving?
- Which books or articles do you want your students to read?
- What are the core communication skills that relates to information giving?
- Who has been the most important expert to drive theory forward in this field?
- What part of information giving theory is most robust in your opinion?

Personal opinion. Example questions:

- What is the most important things you want your students to learn about information giving?
- Which tips or tricks do you most frequently give to students?
- What do students usually struggle with when it comes to information giving?
- What do patients want from doctors when it comes to information giving?
- What are the most important skills of information giving in your opinion?
- If you could teach one thing to all finished doctors and health personnel about information giving or communication in general, what would it be?

Conclusion. Example questions:

- What is your advice for us as we are about to create a new program for information giving for medical students in Oslo?
- What are the pitfall of designing a course on information giving?
- How should we organize teaching of information giving?
- How can we make our new program for information giving as good as possible?

# Consent form

# Request to participate in “Teaching information giving. A qualitative study using in depth interviews of world-leading experts.”

As a leading expert in clinical communication we invite you to participate in a single interview for research during 2022.

**Purpose of the study:**

We are developing an evidence-based program for teaching “information giving” to medical students at the University of Oslo. As a part of this project, we will conduct qualitative semi-structured interviews to gather “know-how” from leading experts in clinical information giving. We hope to distill the expertise of the best professors, teachers and clinicians across the globe to move the field of information giving forwards.

**About the experts:**

We use chain referral sampling for recruitment, where we identify experts in the field from our network and ask for opinion as to who else may be relevant to interview. These experts may be connected to universities, health care organizations or other research organizations with teaching programs in clinical communication. We aim to interview at least 15 experts. Participation is voluntary, and you can withdraw at any time.

**About the interview:**

- The interview will be a single conversation conducted and recorded using Zoom or Teams. The conversation will be transcribed and qualitatively analyzed.

- We will use a semi-structured interview guide, focused on uncovering the in-depth knowledge of the individual participants in a systematic manner.

- The interview will last no more than 1 hour.

- The interviewer is Herman Egenberg, who is a M.D and phd-student with experience both from working with patients and from teaching clinical communication skills to students.

**Most important questions:**

- What are the most important skills to teach medical students about giving information?

- How can doctors help patients recall the information they are given?

- How should information giving be taught to students?

- Which, if any, theoretical framework informs your teaching of information giving?

**Concerns on privacy:**

All interviews will be audiorecorded and transcribed. **We would like to acknowledge all the interview participants in the study, and use quotes which will be presented anonymously. Your personal data will be kept together with the transcript.** All data will be stored according to university guidelines for “yellow data”, using secure servers and encrypted external hard disks. They will only be available to project members. The project ends in July 2025 and the data will be stored for 5 years after this.

You have the right and possibility to get insight in, edit and delete any data gathered in your interview. You also have the right to ask for copies or protest the data.

**Contact info:**

Herman Egenberg. Phd student UiO. Phone: +47 45219089 mail: H.e.egenberg@medisin.uio.no

Hanne Cathrine Lie, professor UiO. Phone: +47 22851466 mail: h.c.lie@medisin.uio.no

Personal data protector (personvernombud) Roger Markraf Bye. mail: personvernombud@uio.no

**Consent for participation in research project:**

“Teaching information giving. A qualitative study using in depth interviews of world-leading experts.”

I have received the information, agree to participate and consent to my name and quotes being used in the study.

Participant name:_______________________________________________________

Date:______________

Participant Signature:____________________________________________________

**COREQ (COnsolidated criteria for REporting Qualitative research) Checklist**

A checklist of items that should be included in reports of qualitative research. You must report the page number in your manuscript where you consider each of the items listed in this checklist. If you have not included this information, either revise your manuscript accordingly before submitting or note N/A.

| **Topic** | **Item No.** | **Guide Questions/Description** | **Reported on**  **Page No.** |
| --- | --- | --- | --- |
| **Domain 1: Research team and reflexivity** | | | |
| *Personal characteristics* | | | |
| Interviewer/facilitator | 1 | Which author/s conducted the interview or focus group? | Methods |
| Credentials | 2 | What were the researcher’s credentials? E.g. PhD, MD | Methods |
| Occupation | 3 | What was their occupation at the time of the study? | Methods |
| Gender | 4 | Was the researcher male or female? |  |
|  |  |  | N/A |
| Experience and training | 5 | What experience or training did the researcher have? | Methods |
| *Relationship with*  *participants* | | | |
| Relationship established | 6 | Was a relationship established prior to study commencement? | N/A |
| Participant knowledge of  the interviewer | 7 | What did the participants know about the researcher? e.g. personal  goals, reasons for doing the research |  |
|  |  |  | Appendix |
|  |  |  |  |
| Interviewer characteristics | 8 | What characteristics were reported about the inter viewer/facilitator?  e.g. Bias, assumptions, reasons and interests in the research topic |  |
|  |  |  | Appendix |
|  |  |  |  |
| **Domain 2: Study design** | | | |
| *Theoretical framework* | | | |
| Methodological orientation and Theory | 9 | What methodological orientation was stated to underpin the study? e.g. grounded theory, discourse analysis, ethnography, phenomenology,  content analysis |  |
|  |  |  | Methods |
|  |  |  |  |
| *Participant selection* | | | |
| Sampling | 10 | How were participants selected? e.g. purposive, convenience,  consecutive, snowball |  |
|  |  |  | Methods |
|  |  |  |  |
| Method of approach | 11 | How were participants approached? e.g. face-to-face, telephone, mail,  email |  |
|  |  |  | Methods |
|  |  |  |  |
| Sample size | 12 | How many participants were in the study? | Methods |
| Non-participation | 13 | How many people refused to participate or dropped out? Reasons? | Methods |
| *Setting* | | | |
| Setting of data collection | 14 | Where was the data collected? e.g. home, clinic, workplace | N/A |
| Presence of non-  participants | 15 | Was anyone else present besides the participants and researchers? |  |
|  |  |  | N/A |
|  |  |  |  |
| Description of sample | 16 | What are the important characteristics of the sample? e.g. demographic  data, date |  |
|  |  |  | Table 1 |
|  |  |  |  |
| *Data collection* | | | |
| Interview guide | 17 | Were questions, prompts, guides provided by the authors? Was it pilot  tested? | Appendix |
|  |  |  |  |
| Repeat interviews | 18 | Were repeat inter views carried out? If yes, how many? | no |
| Audio/visual recording | 19 | Did the research use audio or visual recording to collect the data? | Methods |
| Field notes | 20 | Were field notes made during and/or after the inter view or focus group? | no |
| Duration | 21 | What was the duration of the inter views or focus group? | Methods |
| Data saturation | 22 | Was data saturation discussed? | N/A |
| Transcripts returned | 23 | Were transcripts returned to participants for comment and/or correction? | No |

| **Topic** | **Item No.** | **Guide Questions/Description** | **Reported on**  **Page No.** |
| --- | --- | --- | --- |
|  |  | correction? |  |
| **Domain 3: analysis and**  **findings** | | | |
| *Data analysis* | | | |
| Number of data coders | 24 | How many data coders coded the data? | Table 2 |
| Description of the coding  tree | 25 | Did authors provide a description of the coding tree? |  |
|  |  |  | N/A |
|  |  |  |  |
| Derivation of themes | 26 | Were themes identified in advance or derived from the data? | Table 2 |
| Software | 27 | What software, if applicable, was used to manage the data? | Methods |
| Participant checking | 28 | Did participants provide feedback on the findings? | No |
| *Reporting* | | | |
| Quotations presented | 29 | Were participant quotations presented to illustrate the themes/findings?  Was each quotation identified? e.g. participant number |  |
|  |  |  | Findings |
|  |  |  |  |
| Data and findings consistent | 30 | Was there consistency between the data presented and the findings? | Findings |
| Clarity of major themes | 31 | Were major themes clearly presented in the findings? | Findings |
| Clarity of minor themes | 32 | Is there a description of diverse cases or discussion of minor themes? | Findings |

Developed from: Tong A, Sainsbury P, Craig J. Consolidated criteria for reporting qualitative research (COREQ): a 32-item checklist for interviews and focus groups. *International Journal for Quality in Health Care*. 2007. Volume 19, Number 6: pp. 349 – 357

**Once you have completed this checklist, please save a copy and upload it as part of your submission. DO NOT include this checklist as part of the main manuscript document. It must be uploaded as a separate file.**
